# Combining propensity score methods with variational autoencoders for generating synthetic data in presence of latent sub-groups

**DOI:** 10.1186/s12874-024-02327-x

**Published:** 2024-09-09

**Authors:** Kiana Farhadyar, Federico Bonofiglio, Maren Hackenberg, Max Behrens, Daniela Zöller, Harald Binder

**Affiliations:** 1 Institute of Medical Biometry and Statistics, University of Freiburg, Freiburg, Germany; 2 Freiburg Center for Data Analysis and Modeling, University of Freiburg, Freiburg, Germany; 3 National Research Council of Italy, ISMAR, Forte Santa Teresa, Lerici, Italy

**Keywords:** Synthetic data, Complex distribution, Propensity score, Deep generative model, Variational autoencoder

## Abstract

In settings requiring synthetic data generation based on a clinical cohort, e.g., due to data protection regulations, heterogeneity across individuals might be a nuisance that we need to control or faithfully preserve. The sources of such heterogeneity might be known, e.g., as indicated by sub-groups labels, or might be unknown and thus reflected only in properties of distributions, such as bimodality or skewness. We investigate how such heterogeneity can be preserved and controlled when obtaining synthetic data from variational autoencoders (VAEs), i.e., a generative deep learning technique that utilizes a low-dimensional latent representation. To faithfully reproduce unknown heterogeneity reflected in marginal distributions, we propose to combine VAEs with pre-transformations. For dealing with known heterogeneity due to sub-groups, we complement VAEs with models for group membership, specifically from propensity score regression. The evaluation is performed with a realistic simulation design that features sub-groups and challenging marginal distributions. The proposed approach faithfully recovers the latter, compared to synthetic data approaches that focus purely on marginal distributions. Propensity scores add complementary information, e.g., when visualized in the latent space, and enable sampling of synthetic data with or without sub-group specific characteristics. We also illustrate the proposed approach with real data from an international stroke trial that exhibits considerable distribution differences between study sites, in addition to bimodality. These results indicate that describing heterogeneity by statistical approaches, such as propensity score regression, might be more generally useful for complementing generative deep learning for obtaining synthetic data that faithfully reflects structure from clinical cohorts.

## Introduction

There has been a surge of interest in methods for generating synthetic datasets based on real clinical data [1]. Such approaches may, e.g., be useful for providing data protection when even heavily sampled anonymized datasets do not meet privacy standards [2]. In addition to the application for single datasets, another usage scenario is in federated computing platforms, such as DataSHIELD [3], for simultaneously generating synthetic data at several sites and then pooling the synthetic data for test-driving analyses (e.g., [4] or our own proposal in [5]). Beyond these data protection use cases, synthetic data can also be used for oversampling minority classes [6] or, more generally, augmenting the data (e.g., [7, 8]). Furthermore, simulation studies and in silico clinical trials can benefit [9–12].

When using such techniques for clinical cohort data from observational studies, or also from randomized trials, faithful handling and potential preservation of heterogeneity across patients is important, in particular concerning sub-group structure. The importance of sub-groups in a clinical setting is reflected in a long history of research on biases that can arise when ignoring sub-group structure, e.g., as in Simpson’s paradox [13]). Furthermore, there is a multitude of approaches for dealing with sub-group effects, such as propensity scores for properly assessing treatment effects [14], and also for more generally combining groups in clinical cohorts (e.g., our results in [15]). Therefore, it might also be attractive to complement synthetic data techniques with approaches such as propensity score regression for handling heterogeneity due to known sub-groups. In addition to proposing a corresponding approach, we will also address heterogeneity due to unknown sub-groups. In clinical settings the known sub-groups may consist of unknown sub-groups, e.g., different severity of the disease. Contrary to the known sub-groups, which have explicit labels, the unknown ones are only reflected in the distributions of different variables. Therefore, one of the methods to check for the presence of unknown sub-groups is the use of descriptive statistics and visualization methods, which can reveal the potential presence of sub-groups. It includes examining the distribution of predictor variables and looking for patterns that suggest multiple underlying patterns that might not be obvious in aggregated data. For instance, specific sub-groups might have distinct frequencies or relationships between categorical variables, or certain distributions like skewed and bimodal might suggest the presence of an unknown sub-group [16]. Therefore, we complement synthetic data techniques with pre-transformations to preserve the unknown structures and recover the bimodal or skewed distributions of continuous covariates. Moreover, adapting our approach, we consider relationships between continuous and binary variables to reproduce the characteristics of unknown sub-groups in the synthetic data.

The challenge of properly handling sub-groups already becomes apparent when considering one of the most prominent techniques for synthetic data generation, namely generative adversarial networks [17]), which had initially been developed for image data. There, the price for generating crisp synthetic images seems to be mode collapse, where certain sub-groups of the original dataset are no longer reflected [18]. Therefore, we consider an alternative popular technique as the basis for our proposed approach, specifically variational autoencoders (VAEs) [19]. For modeling the relationships between multiple variables in a given dataset, VAEs build on an underlying low-dimensional latent representation, where artificial deep neural networks are used for estimating conditional distributions. The latter are amenable for combination with propensity scores obtained from regression models involving sub-group labels.

**Fig. 1:**
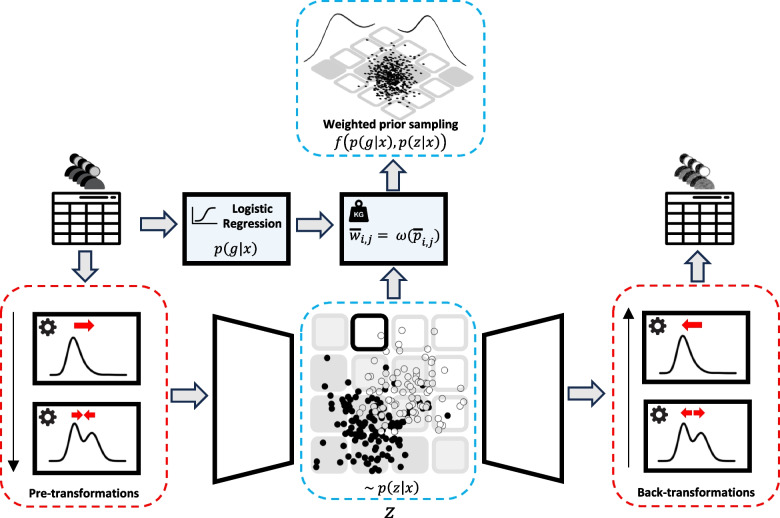
Schematic overview of the proposed VAE-based approach, consisting of two primary components: (1) Unknown sub-groups within heterogeneous distributions are addressed through pre-transformations, as indicated by model components in red boxes. (2) Known sub-groups are handled using propensity score modeling *p*(*g*|*x*) and weighted prior sampling, indicated by blue boxes. The function $\omega$ incorporates the weights based on estimated propensity scores shown in Eqs. (14) or (15)

However, VAEs have also been developed with image data in mind, where some homogeneity in distributions can be assumed [20]. This is reflected in an underlying assumption of a Gaussian prior on the latent representation, and thus VAEs have limitations with data deviating from unimodal symmetric distributions. While VAE-based approaches already exist for addressing data diverging from normal distributions based on modifying the prior on the latent representation (e.g., [21]), these are not flexible enough when different variables in the original data exhibit different kinds of peculiarities in their distribution. This motivates our pre-transformation component at the level of the original variables in our proposal.

There are also proposals for synthetic data outside the deep neural network community, e.g., using sampling based on the correlation matrix [22]. Similarly, we have introduced an approach based on Gaussian copula together with simple non-disclosive summaries [23]. While we will consider the latter for performance comparison, our focus is on VAEs as their latent representations provide a starting point for complementing information from propensity score approaches. Figure 1 shows the schematic overview of our approach.

In this study, we introduce a deep learning arechitecture to generate synthetic data in presence of sub-groups. To achieve this, we integrate the propensity score concept with and adapted version of VAE, a combination that, to the best of our knowledge, has not previously been explored. “Methods” section introduces the proposed approach, specifically highlighting how heterogeneity due to known and unknown sub-group structures is handled. “Evaluation of the method for unknown sub-group structures” section contrasts our pre-transformation-enhanced VAE with other techniques in a simulation study and real data from a stroke trial. “Evaluation of the method for known sub-group structures” section presents the combination of propensity scores with the latent representation of VAEs for simulation data, and weighted sampling is illustrated for the stroke trial example. We conclude with a discussion in “Discussion” section. Source code for our approach is available on GitHub.

## Methods

### General framework

Encoding data into a latent space allows for a better understanding of the data structure by revealing patterns not apparent in the original high-dimensional space. Specifically, dimension reduction techniques using two or three dimensions provide a visual insight into the data. If we consider the latent space to be given by a random variable *z*, we can define a model *p*(*z*|*x*) as the probability distribution of the latent space given *x*, which denotes the whole set of observations. As mentioned before, we should consider the heterogeneity between sub-groups in the dataset. In instances where we have known sub-groups with labels, a distinct model can approximate *p*(*g*|*x*) , where *g* is a random variable producing the sub-group label. In this setting, our objective is to formulate a function *f*(*p*(*z*|*x*), *p*(*g*|*x*)) so that we can produce the structure of interest in our generated data (e.g., removing systematic differences or pronouncing one sub-group structure). For the other sub-groups with no explicit label, i.e., where we do not have access to *p*(*g*|*x*) , and *f* would be implemented only based on *p*(*z*|*x*), our goal is to shape a latent structure reflecting the unknown sub-groups and consequently reconstruct the marginal distributions which are indications of existing underlying, yet unrecognized, sub-group structures. In the following sections, we explain different parts of our general framework, including the approximation for *p*(*z*|*x*) using a variational autoencoder, dealing with unknown and known sub-groups and how we implement *f*.

### Variational autoencoders (VAEs)

One of the standard methods to approximate *p*(*z*|*x*) is the use of a specific type of autoencoders called variational autoencoder (VAE). The simplest autoencoders consist of an encoder and a decoder, which are both multi-layer perceptrons, i.e., a neural network with one input layer, one output layer, and one or multiple hidden layers. As shown in Eq. (1), each layer, denoted by *l*, corresponds to a linear combination of its inputs $h^{l-1}$ (which is the output vector of the previous layer or input data if $l = 1$) and weights $w^{(l)}$, and biases $b^{(l-1)}$, followed by a non-linear transformation $g^{(l)}$ called activation function. The layer output is a vector, which is used as input for the next layer.

1 $$\begin{aligned} \textbf{h}^{(l)} = g^{(l)}\left( \textbf{W}^{(l)}\textbf{h}^{(l-1)} + \textbf{b}^{(l)}\right) \end{aligned}$$

The encoder part reduces the dimensions of the input layer to a latent embedding, and the decoder part tries to reconstruct the data from that [24]. A VAE [19] is a probabilistic version of an autoencoder, where the latent representation is considered to be given by a random variable with a prior distribution assumed to be a standard normal distribution. Based on Bayes’ rule, the posterior distribution of the latent variable *z* given the observed variable *x* can be obtained via the Bayes’ rule in Eq. (2). The integral in the denominator of the formula is computationally intractable, even for a relatively low-dimensional *z*. One solution is to use variational inference to approximate *p*(*z*|*x*) by a distribution *q*(*z*|*x*), which is a member of a parametric family of distributions, e.g., a Gaussian distribution with diagonal covariance, which is typically used in VAEs. Then, finding the posterior becomes an optimization problem, i.e., minimizing the Kullback-Leibler (KL) divergence between these two distributions, which can be calculated as shown in Eq. (3).

2 $$\begin{aligned} p(z|x)=\frac{p(z,x)}{p(x)}=\frac{p(z,x)}{\int p(z,x) dz} \end{aligned}$$

3 $$\begin{aligned} D_{K L}(q \Vert p)=E_{q(z|x)}[\textrm{log} (q(z|x))-\textrm{log} (p(x, z))]+\textrm{log} \left( p(x)\right) \end{aligned}$$

Since $\textrm{log} \left( p_{x}(x)\right)$ in Eq. (3) is constant, and the KL divergence is a positive value, minimizing it is equivalent to maximizing the so-called evidence lower bound (ELBO) $E_{q(z|x)}[\textrm{log} (q(z|x))-\textrm{log} (p(x, z))]$. In the VAE, the encoder models $q_{\varphi }(z |x)$, where the parameters $\varphi$ are the encoder weights and biases, and the decoder models $p_{\theta }(x |z)$, with parameters $\theta$. The ELBO can be rewritten to obtain the loss function, shown in Eq. (4), optimizing $\varphi$ and $\theta$. The first term on the right-hand side corresponds to reconstruction loss. The second term is the Kullback-Leibler divergence between the approximated posterior and the prior distribution.

4 $$\begin{aligned} \textrm{loss}\left( x_{i}\right) =-E_{q_{\varphi }\left( z|x_{i}\right) }\left[ \textrm{log} p_{\theta }\left( x_{i}|z\right) \right] + D_{K L}\left( q_{\varphi }\left( z|x_{i}\right) || p_{\theta }(z)\right) \end{aligned}$$

There are two methods for obtaining synthetic data from a trained VAE: 1) sampling *z* from the approximated posterior given the original data or 2) sampling *z* from the standard normal distribution (prior), followed in both cases by using the obtained values of *z* as input for the decoder. The latter can better preserve the privacy because the original data can influence the synthetic data only via the trained parameters of the decoder. Therefore, if the VAE is not overfitted, having a low-dimensional latent space and sampling from prior can decrease the risk of data leakage.

### Dealing with unknown sub-groups

To preserve the unknown sub-group structure, we aim to faithfully recover the marginal distributions. In this work, we concentrate on reconstructing Bernoulli (for binary variables), skewed and bimodal distributions. First, we need a VAE architecture to generate both continuous and binary variables (“VAE for combining continuous and binary variables” section). Then, we use pre-transformations to transform the original data to remove skewness and bimodality so that a VAE can better model it. As is common in machine learning, to speed up the VAE training, we scale the data between zero and one. This needs to be considered in the backward process as well, i.e., after getting the output from the VAE, we have to transform the output back. Figure 1 shows how the pre-transformation steps are incorporated into the general framework. The pre-transformation for skewed distributions is explained in “Box-Cox transformation” section, and the pre-transformation for bimodal distributions is described in “Transformation for bimodality” section.

#### VAE for combining continuous and binary variables

To generate both continuous and binary variables, we use an architecture with separate parts corresponding to the two variable types. For a decoder with $l+1$ layers, hidden layer $h_{D}^{(l)}$ serves as the joint basis for continuous and binary covariates, e.g., for representing correlation patterns between the two types of variables. Then, in the next layer, we have a group of neurons denoted by $\mu _{D}$ and $\sigma _{D}$ for the continuous variables and a group of neurons represented by $\pi _{D}$ for the binary variables. This means that the reconstructed values for the continuous variables are subsequently sampled from $N\left( \mu _{D}(h_{D}^{(l)}(z)), \sigma _{{D}}(h_{D}^{(l)}(z))\right)$ and for the binary variables by sampling from Bernoulli $(\pi _{D}(h_{D}^{(l)}(z)))$. Assuming $x_{i, j}$ as the *j*-th continuous variable of $x_{i}$ and $x_{i, k}$ as the *k*-th binary variable of $x_{i}$ and for *x* with $p_{c}$ continuous variables and $p_{b}$ binary variables, the loss function can be computed by Eq. (5). The parameters of VAE are the weights and biases of the encoder and decoder $(\varphi , \theta )$.

5 $$\begin{aligned} \begin{array}{ll} \textrm{loss}\left( x_{i}\right) = & -\sum \limits _{k=1}^{p_{b}} \textrm{logpdf} \left( \textrm{Bernouli}\left( \pi _{D_{k}}(h_{D}^{(l)}(z_{i}))\right) , x_{i, k}\right) \\ & -\sum \limits _{j=1}^{p_{c}} \textrm{logpdf}\left( \textrm{Normal}\left( \mu _{D_{j}}(h_{D}^{(l)}(z_{i})),\sigma _{D_{j}}(h_{D}^{(l)}(z_{i}))\right) , x_{i, j}\right) \\ & +D_{K L}\left( q_{\varphi }\left( z|x_{i}\right) \Vert p_{\theta }(z)\right) . \end{array} \end{aligned}$$

With this modification, we can generate both binary and continuous variables, and thus cover the heterogeneity in the data types. However, another well-known problem when having different data types, e.g., binary and continuous or different sources, e.g., image and tabular data, is data fusion. There are different ways to tackle this (introduced in [25]). Given that there is no universally superior method for data fusion, we try two strategies as a hyper-parameter in each of our experiments. In this work, we investigate the early fusion, i.e., concatenation of the data type from the beginning, or late fusion, i.e., having two different encoders for binary and continuous variables.

#### Box-Cox transformation

To remove the skewness, we use a family of power transformations called the Box-Cox transformation, shown in Eq. (6), suggested by [26]. In this formula, $\lambda _{2}$ is a shifting value to make the data positive, and $\lambda _{1}$ is the main parameter of the transformation. To estimate $\lambda _{1}$, we minimize the negative log-likelihood of the transformed values using gradient descent using Eq. (7). In this optimization problem, we try to find the local minimum of this criterion using the gradient concept. After getting the output from the VAE, we have to transform the data back. The back-transformation for Box-Cox transformation is shown in Eq. (8).

6 $$\begin{aligned} f_{\text {BoxCox}}\left( x ; \lambda _{1}, \lambda _{2}\right) = \left\{ \begin{array}{ll} \frac{\left( x+\lambda _{2}\right) ^{\lambda _{1}}-1}{\lambda _{1}} & \ \lambda _{1} \ne 0 \\ \textrm{ln} \left( x+\lambda _{2}\right) & \ \lambda _{1}=0 \end{array}\right. \end{aligned}$$

7 $$\begin{aligned} \begin{array}{c} L(\lambda _1, \lambda _2 | x) = -\frac{N}{2} \textrm{log}(\sigma ^2 + \epsilon ) + (\lambda _1 - 1) \sum \limits _{i=1}^{N} \textrm{log}(x_i + \lambda _2 + \epsilon ) \\ \text {where}\\ \quad \sigma ^2 = \text {Var} ( f_{\text {BoxCox}}\left( x ; \lambda _{1}, \lambda _{2}\right) ) \end{array} \end{aligned}$$

8 $$\begin{aligned} f^{-1}_{\text {BoxCox}}(y) = \left\{ \begin{array}{ll} ^{\lambda _1}\!\!\!\sqrt{\lambda _1 y^{(\lambda )} + 1} - \lambda _2, & \ \lambda _1 \ne 0 \\ e^{y(\lambda )} - \lambda _2, & \ \lambda _1 = 0 \end{array}\right. \end{aligned}$$

#### Transformation for bimodality

The second transformation aims to make a bimodal distribution closer to an unimodal one by bringing the peaks closer and keeping the shape of the tails close to a normal distribution. We use a power function $x^{\rho }$ with the odd integer $(\rho =2 k+1$ with $k=1,\ldots , N)$, and this will work if we can shift and scale values such that the two peaks of the bimodal distribution fall within $(-1,1)$. Therefore, we must find the best values for the shifting parameter $\alpha$, positive scaling parameter $\beta ^2$, and power $\rho$. to be able to continuously differentiate w.r.t these parameters for gradient-based optimization, we use $\textrm{sgn}(x)|x|^{\rho }$, so that we have the same behavior for all different values of $\rho$. Hence, our transformation will be as Eq. (9).

9 $$\begin{aligned} f(x) = \textrm{sgn}(\frac{(x + \alpha )}{\beta ^2} ) |\frac{(x + \alpha )}{\beta ^2} |^{\rho } \end{aligned}$$

For parameter optimization, we need a criterion that reflects closeness to an unimodal distribution. We considered maximum likelihood and the bimodality coefficient ($\left( b=\frac{\gamma ^{2}+1}{\kappa }\right.$ where $\gamma$ is the skewness and $\kappa$ is the kurtosis), which both did not give adequate results as they decreased the variance too strongly. Therefore, we minimize a 1-sigma criterion, shown in Eq. (10), to optimize the parameters. In this Equation, $Q_\tau (x)$ represents the $\tau$-th percentile of *x*. This optimization problem requires careful initialization of the parameters since the 1-sigma criterion is only a proxy for the deviation from an unimodal distribution. First, to have $\rho > 1$, we define the power parameter as $\rho = 1 + pow^2$. We start with $pow = 0$ and $\beta ^ 2 = 1$ for the scaling parameter to keep the data unchanged if it is normal/unimodal. Furthermore, finding the valley between two peaks in a heuristic way is a good starting point for the shifting variable ($\alpha$). We use an iterative heuristic algorithm based on kernel density estimation to initialize this value. In this method, we start estimating the density function with a very small bandwidth and find the local maxima of the function. Consequently, we gradually increase the bandwidth and continue until we only have a limited number of peaks (e.g., five). Then, we pick the two highest peaks and the deepest valley between these two. The value of the valley can be set as the initial value of $\alpha$.

10 $$\begin{aligned} 1-\textrm{sigma}_{\text {criterion}}(x) = \left| Q_{0.84}(x) - Q_{0.5}(x) - \sigma _{x} \right| + \left| Q_{0.5}(x) - Q_{0.16}(x) - \sigma _{x} \right| \end{aligned}$$

Like the first transformation, we also need the back transformation function for the second one. The reverse function is shown in Eq. (11). Applying the pre-transformations addresses the challenge of heterogeneity in the distributions of continuous variables.

11 $$\begin{aligned} f^{-1}(y) = \beta ^2 y^{\frac{1}{\rho }} \textrm{sgn}(y) - \alpha \end{aligned}$$

### Dealing with known sub-groups

#### Propensity score estimation

Dealing with known sub-groups requires an approach that generates the structure of interest, i.e., removing the systematic differences between sub-groups or pronouncing the characteristics specific to one sub-group. To sample from areas of the latent space which have our structure of interest, we need a quantitative guide, such as *p*(*g*|*x*) , to be used as a weighting system when sampling from the prior distribution of *z*. Therefore, we build a model for estimation of *p*(*g*|*x*) to predict the sub-group membership for each observation $x_i$. To achieve this, we use propensity scores, i.e., the probability of an observation belonging to a group given a set of covariates [27]. We use a logistic regression for the binary classification of sub-groups in our datasets outlined in “Datasets and results” section. The model prediction for a data point $x_i$ will then be the probability of $x_i$ belonging to the sub-group number one, i.e., $p(g=1|x = x_i)$, where $g \in \{1, 2\}$. We use the original data space for propensity score estimation for two main reasons. First, this approach allows us to leverage the rich information inherent in the original data, which can be crucial for accurate propensity score estimation. Second, logistic regression is effective in the original data space, even when faced with complex data distributions. This model robustness might not hold in a reduced-dimensional latent space. In such lower-dimensional spaces, the simplification of data can lead to a loss of important information, especially when dealing with heterogeneous and complex distributions.

#### Propensity score-based sampling method

We can use the propensity score concept for assigning weights to different areas of the latent space learned by the VAE. To see whether we can use propensity scores concept as a guide for sampling from the latent space, after training the VAE, we visualize the latent space with the propensity score values. For this, we divide the latent space into a grid of cells with a tenable size *d*. For a two-dimensional latent space $z=((z)_1,(z)_2)$, we then define a matrix *A*, where $A_{i,j}$ is a cell in the grid on the latent space. In this grid, *i* denotes the index of the cell along the $(z)_1$-axis, while *j* denotes the index of the cell along the $(z)_2$-axis. Therefore, for each cell $A_{i,j}$ we have that for all $z\in A_{i,j}$:

12 $$\begin{aligned} \begin{array}{l} \underset{z}{\textrm{min}}(z)_1< (z)_1< (\underset{z}{\textrm{min}}(z)_1 + (i-1) \cdot d), \\ \quad \text {for } i = 1, \ldots , N_1 = \left\lceil \frac{\max _z(z)_1 - \textrm{min}_{z}(z)_1}{d} \right\rceil , \\ \underset{z}{\textrm{min}}(z)_2< (z)_2 < (\underset{z}{\textrm{min}}(z)_2 + (j-1) \cdot d), \\ \quad \text {for } j = 1, \ldots , N_2 = \left\lceil \frac{\max _z(z)_2 - \textrm{min}_{z}(z)_2}{d} \right\rceil . \\ \end{array} \end{aligned}$$

After making a grid on the latent space, we need to calculate the propensity score for each cell. For this, we fit a logistic regression on the observations $x_{1 \ldots n}$, and then we calculate the propensity score using the predictions of a logistic regression model. After this, each point in the latent space of VAE $z_k$, which is the mapping of an observation $x_k$, has a propensity score $p_{x_k}$. Then, we calculate the propensity score for each cell, averaging the propensity score of the points that are in that specific cell. This is shown in Eq. (13).

13 $$\begin{aligned} \bar{p}_{i,j} = \frac{1}{n} \sum \limits _{k=1}^{n} p_{x_k} \cdot \mathbb {I}(z_k \in A_{i,j}) \end{aligned}$$

After the propensity score calculation, as shown in Fig. 1 we can overlay the grid with the scatter plot of the latent space, color-coded by the existing sub-groups in our dataset. If the cells in the grid, colored by propensity score, correspond to the color of the majority group of the points in each cell, we can use this as a guide for sampling from the prior distribution, i.e., we can define weights based on the scenario in which we want to generate synthetic data. For this, we use Inverse Probability of Treatment weighting (IPTW) [28] to define a new weighting approach for our scenario. Suppose we only want to generate individuals that are common for both sub-groups. In that case, we can use Eq. (14). If we want only to have individuals with the characteristics of one group, say, where $g=0$, and individuals should have a small value of $\bar{p}_{i,j}$, we can use the weighting system shown in Eq. 15. In both equations, $\delta$ denotes the acceptable deviation from $\bar{p}_{i,j} = 0.5$, representing the areas common for both populations. This value can be tuned as a hyperparameter. In this work, we tried $\delta = 0.05, 0.1, 0.2$. A key consideration in selecting this hyperparameter is the degree of overlap among sub-groups in the latent space, i.e., the more overlap, the larger the value for $\delta$. Even though the method is described for two-dimensional latent space, this method is generalizable to any size of latent space dimensions as long as we can avoid the curse of the dimensionality problem, i.e., the number of points in the grid cells is not very sparse. However, in the clinical settings we discuss in this work, the number of variables is not very large to necessitate going for a higher-dimensional latent space. Moreover, for the visualization part, we can always use dimensionality reduction techniques like principle component analysis (PCA) to be able to overlay the latent structure and the propensity score based guide.

14 $$\begin{aligned} w_{i,j} = \left\{ \begin{array}{ll} 0 & \ \left| {\bar{p}_{i,j} - 0.5}\right|> \delta \\ \frac{1}{\bar{p}_{i,j}} & \ \bar{p}_{i,j}>0.5 \\ \frac{1}{1 - \bar{p}_{i,j}} & \ \bar{p}_{i,j}<0.5 \end{array}\right. \end{aligned}$$

15 $$\begin{aligned} w_{i,j} = \left\{ \begin{array}{ll} 0 & \ \bar{p}_{i,j} > 0.5 + \delta \\ \frac{1}{\bar{p}_{i,j}} & \ \bar{p}_{i,j} \le 0.5 + \delta \end{array}\right. \end{aligned}$$

We obtain weights for each cell using Eqs. (14) or Eq. (15) and subsequently normalize them, as shown in Eq. (16).

16 $$\begin{aligned} \bar{w}_{i,j} = \frac{w_{i,j}}{\sum _{i=1}^{N_1} \sum _{j=1}^{N_2} w_{i,j}} \end{aligned}$$

We can now use propensity scores as a guide for prior sampling to generate a synthetic dataset. Specifically, we sample from the prior distribution, *N*(0, 1), then find the corresponding cell and the weight assigned to that cell. Next, we sample from a Bernoulli distribution with $P(X = 1) = \bar{w}_{i,j}$. This step is the decision flag to include or reject the sampled value. Repeating the process until we reach the intended sample size gives us a set of samples to feed to the decoder and get the output as our synthetic dataset. Note that the weighted sampling is performed in the data generation step, and in the training phase of VAE, we just use the posterior sampling.

## Evaluation of the method for unknown sub-group structures

### Baseline approaches

To evaluate our method in dealing with unknown sub-groups, we compare the utility of synthetic data generated with the standard VAE [19] with the minor modifications in encoder and decoder (without pre-transformations), the VAE with an autoregressive implicit quantile network (AIQN) [29] (called QVAE here), generative adversarial networks (GAN) [17] and NORTA-J, our Gaussian copula-based approach with first four moments [23]. For simplicity, we use the same architecture for all the VAE-based approaches. The evaluation metrics are explained in “Approaches for comparison and evaluation criteria” section.

In the QVAE approach, quantile regression allows for more flexibility in the VAE latent space. Specifically, a neural network embedded in the latent space implements the quantile regression for each dimension. For each data point $x_i$, we get a $z_i$ in the latent space. We use a random number $0.05< \tau <0.95$ as an input of each quantile network, and then for each dimension *k*, we use the $\tau$ and $z_{i_1}, \ldots , z_{i_{k-1}}$ as the input and $z_{i_k}$ as the output. This means that for the first dimension, the network has one input, i.e., $\tau$, and one output, i.e., $z_1$. Because we have a conditional network based on the previous dimensions and $\tau$, we need to use the best order of $z_{i_{1, \ldots , l}}$ for the quantile network architecture. We use the Kolmogrov-Smirnov test to determine which order makes the conditional distribution closer to a normal distribution. We train the network with the quantile regression loss function for each dimension. For more information on the details of the QVAE approach, see [29].

GANs comprise two multiple-layer perceptrons, called the discriminator and the generator. The generator part is responsible for generating synthetic data, and the discriminator aims to distinguish between real data and generated data. The better the generator, the harder it is for the discriminator to distinguish real and generated data. After training the generator to fool the discriminator, which is trained simultaneously, the generator should be able to generate realistic synthetic data. For more information see [17].

The method from our previous work, which we call NORTA-J here, infers the original individual person data (IPD) characteristics from summary statistics. This method generates synthetic data through a Gaussian copula inversion technique known as NORTA, which models the dependency structure of the data variables. The marginal distributions of IPD are constructed using the Johnson system of distributions, parameterized by empirical marginal moments (e.g., mean, variance) and the correlation matrix [23].

Additionally, we consider comparing our approach with a method that involves applying the empirical CDF (cumulative distribution function) followed by a quantile transformation to a normal distribution (i.e., applying the inverse Normal CDF). This method is a non-parametric pre-transformation technique. We refer to it as a quantile pre-transformation approach or QP-VAE. For such transformation, increasing the number of quantiles raises the risk of identification [30, 31]. This issue is especially pronounced for extreme quantiles, which are sensitive to outliers or unique values, potentially exposing information about specific individuals [32]. Nevertheless, we use this approach as a baseline model to show the impact of proper pre-transformations on the data.

### Approaches for comparison and evaluation criteria

As the first quantitative measure to compare synthetic data from the different approaches, we use a utility metric $\psi$, proposed by Karr et al. [33] and extended by Snoke et al. [34]. The idea behind this metric is that if a synthetic dataset has a high quality in terms of utility, a classification model cannot distinguish the synthetic samples from real observations well. This means that, ideally $p(x_i \in S_{\textrm{syn}}) \sim p(x_i \in S_{orig})$, where $S_{\textrm{syn}}$ is the synthetic dataset and $S_{\textrm{orig}}$ is the original dataset. Therefore, if we can show the probability of being a member of the synthetic dataset is around 0.5, we can claim that synthetic and original datasets have similar distributions. Therefore, we combine these two datasets, and add a label variable $y_i$, where $y_i = 1$ if ($x_i \in S_{syn}$) and $y_i = 0$ if ($x_i \in S_{\textrm{orig}}$). Following this, we apply the Classification and Regression Tree (CART) method to construct a decision tree. We choose the CART model because it excels in scenarios where the original dataset deviates from a normal distribution due to its ability to form decision boundaries in complex, non-linear data spaces. Using this fitted model, we can predict $\widehat{y}_{i}$ for $x_{i=1,\ldots N}$ where $N = n_{\text {syn }} + n_{\text {orig }}$, which is the probability of each observation belonging to synthetic data. The more $\widehat{y}_{i}$ deviates from the ratio of synthetic data size to the merged data size $\left( c=\frac{n_{s y n}}{N}\right)$, the less similar are original and synthetic data. Hence, this utility metric can be measured as shown in Eq. (17).

17 $$\begin{aligned} \psi =\frac{1}{N} \sum \limits _{i=1}^{N}\left( \widehat{y}_{i}-c\right) ^{2} \end{aligned}$$

Snoke et al. [34] suggested another metric with the same idea, where the null hypothesis is defined by the CART-based classifier trained on the data with true labels performing as random as for permuted labels, i.e., the original dataset is very similar to the synthetic dataset. Based on several permutations of the labels ($y_i$), the value $\psi _{\textrm{perm}_j}$ can then be calculated as in Eq. (17), where $\textrm{perm}_j$ is the *j*-th permutation. We can then calculate the mean over all iterations $\bar{\psi }$ using Eq. (18). We set the number of permutations to 100. Then, the final metric is given by Eq. (19).

18 $$\begin{aligned} \bar{\psi } = \frac{1}{n_{\textrm{perm}}} \sum \limits _{j=1}^{n_{\textrm{perm}}} {\psi }_{\textrm{perm}_{j}} \end{aligned}$$

19 $$\begin{aligned} \psi _{ratio} =\frac{\psi }{\bar{\psi }} \end{aligned}$$

In the second approach, we use visual comparisons of marginal distributions. This way, we can check which methods can reconstruct the marginal distributions and which ones and to what extent fail to do so.

**Table 1:** Comparison of synthetic data generated from simulation data, evaluated by two utility metrics (lower values indicate better performance)

Metric	Methods					
	Our method	NORTA-J	GAN	VAE	QVAE	QP-VAE
$\bar{\psi }$	$0.068 \pm 0.01$	$0.097 \pm 0.01$	$0.156 \pm 0.01$	$0.093 \pm 0.01$	$0.094 \pm 0.00$	$0.057 \pm 0.01$
$\psi _{ratio}$	$1.292 \pm 0.15$	$2.272 \pm 0.23$	$3.008 \pm 0.16$	$1.776 \pm 0.14$	$1.776 \pm 0.08$	$1.153 \pm 0.12$

### Datasets and results

#### Simulation data

To evaluate our method, we use a published realistic simulation design based on a large breast cancer study [35, 36]. Specifically, we use the specification published on Zenodo by [37]. In this simulation study, there is an Exposure variable indicating two different cohorts, i.e., the patients exposed to radiotherapy and non-exposed patients. The outcome of this dataset, denoted as *y*, is defined as having 5-year progression-free survival. The sample size equals 2,500, and there are 21 variables, where 12 are binary, and the rest are continuous variables. Since this simulation data is designed to have real-world distributions, it contains moderate to highly skewed variables. We modify the dataset to additionally include a variable with a bimodal distribution. For this, we generate a bimodal distribution based on the exposure variable by sampling from *N*(0, 1) for $E=0$ and sampling from *N*(4, 1) for $E=1$. The obtained bimodal distribution is also attractive for evaluating our proposed approach because this distribution is not symmetric due to the imbalanced distribution of the exposure variable. On the other hand, we pick the mean of two normal distributions such that the modes are not very far and have overlaps, making it harder for the VAE to imitate the data. As explained above, we optimize the parameters of the pre-transformations and then train the VAE. Table 1 shows the quantitative comparisons described in  “Approaches for comparison and evaluation criteria”. To report uncertainty, we ran all the experiments 10 times in a 10-fold cross-validation approach. Then we report the mean and standard deviation of defined criteria for ten trained models on the heldout data. Running the experiments in the 10-fold cross-validation setting has a limitation for the QP-VAE. When we fit the quantile transformers, the learned quantiles are based on the range and distribution of training set and when we apply this to unseen data (validation or test sets) with values outside that range, it can lead to undefined values. Therefore, we have to clip the out-of-bound values in the range of training set. After doing this, the approaches with pre-transformations show the best performance based on both of these criteria, followed by the NORTA-J approach. The decision trees are fitted with a minimum leaf size of 20 and a maximum depth of 25. With this quantitative measurement QP-VAE shows better results in comparison to our proposed pre-transformation.

**Fig. 2:**
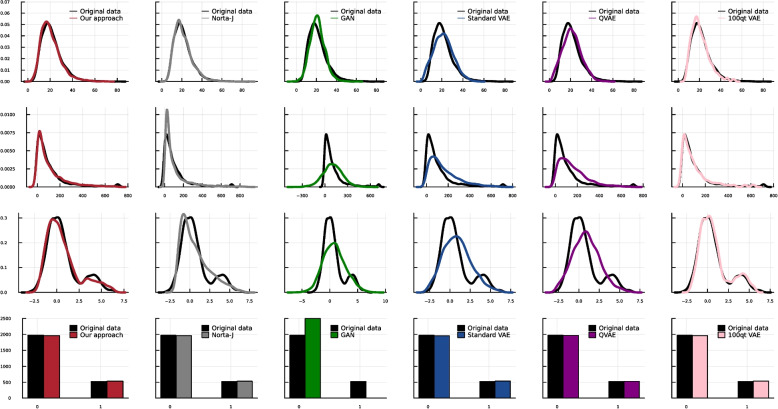
Visual comparisons of marginal distributions in synthetic dataset generated by different methods. In this figure, we show four different variables with different types of distributions. The first row shows the slightly skewed variables, the second is the severely skewed variable, and the third row shows the bimodal variable. The fourth row shows a binary variable. In the columns, different methods are illustrated

In addition to quantitative comparisons, Fig. 2 shows the visual comparisons of the marginal density diagrams and histograms of selected variables. In this step, we used all the data points to generate the synthetic data. Here, we show three exemplary continuous variables (slightly skewed, severely skewed, and bimodal) generated by different methods in comparison to the original data and the histograms of a binary variable. As illustrated, our method can generate both slight and severe skewness in the data, i.e., first and second rows. This is when the standard VAE and QVAE methods cannot reconstruct the skewness as realistically as the original data. Looking at the generated marginal distributions with QP-VAE, we see that none of the tails of the distributions are well reconstructed, and we have in particular undesirable patterns in the tails of skewed distribution. The trained GAN also fails to reproduce the skewness. Norta-J can perfectly reconstruct the slight skewness, but when it comes to severe skewness, our approach outperforms it with respect to the mode and range of data. In addition, the pre-transformation VAEs are the only ones that can reconstruct the bimodality, and still, the QP-VAE approach fails in the reconstruction of the range of data. For GANs, the problem of mode collapse makes synthetic data generation with bimodality more complicated, in particular, when there are different unknown sub-groups. It is worth mentioning that we use different sets of hyperparameters for the deep learning-based approaches, and we pick the most robust results.

**Table 2:** Comparison of synthetic data generated from IST data, evaluated by two utility metrics (lower values indicate better performance)

Metric	Methods					
	Our method	NORTA-J	GAN	VAE	QVAE	QP-VAE
$\bar{\psi }$	$0.091 \pm 0.03$	$0.085 \pm 0.04$	$0.114 \pm 0.01$	$0.106 \pm 0.03$	$0.094 \pm 0.03$	$0.041 \pm 0.00$
$\psi _{ratio}$	$2.449 \pm 0.94$	$2.707 \pm 1.15$	$3.084 \pm 0.25$	$2.85 \pm 0.92$	$2.525 \pm 0.82$	$1.277 \pm 0.11$

**Fig. 3:**
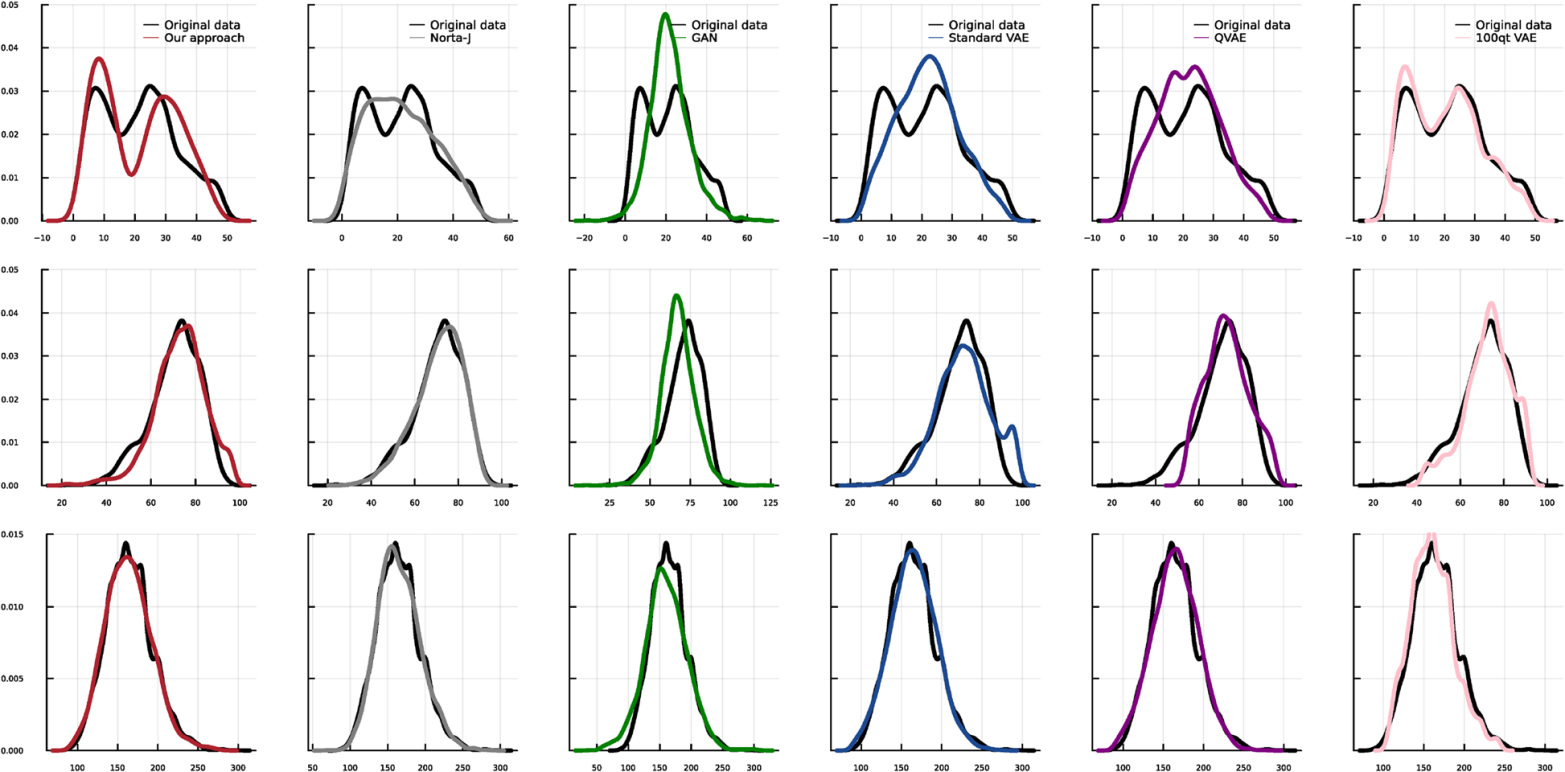
Visual comparisons of marginal distributions in synthetic dataset generated by different methods. This figure shows three continuous variables, including a bimodal distribution, i.e., shown in the first row. In the columns, different methods are illustrated

#### Real data

For a real data evaluation, we consider the IST dataset, which originates from a large international multi-center clinical trial for stroke patients [38] and was also used in our other work in [23]. Specifically, we use a subset of variables, including randomization variables, i.e., conscious state (RCONSC $=$ drowsy, unconscious or alert), the delay between stroke and randomization (RDELAY in hours), gender (SEX $=$ male/female), AGE, RSLEEP (symptoms noted on waking yes/no), atrial fibrillation (RATRIAL$=$ yes/no), CT before randomization (RCT $=$ yes/no), infarct visible on CT (RVISINF $=$ yes/no), heparin with 24 hours prior to randomization (RHEP24 $=$ yes/no), aspirin with three days prior to randomization (RASP3 $=$ yes/no), systolic blood pressure (RSBP), trial aspirin allocated (RXASP $=$ yes/no, trial heparin allocated (RXHEP $=$ yes/no). We exclude the other randomization variables because of the high proportion of missing values. In addition to this, we used FDEAD, i.e., the outcome defined as dead at six-month follow-up. We also added COUNTRY and derived the REGION (EU-EAST, EU-NORTH, EU-WEST, and EU-SOUTH) from that, to have labels for known sub-groups in the data for using the propensity score-based approach. Excluding the individuals with missing values and those in EU-WEST and EU-SOUTH, we create a rather small dataset with 2,668 records. Among these features, blood pressure, age, and the RDELAY are continuous, the level of consciousness is categorical (with three different values), and the rest are binary. The variable RDELAY has bimodality. We change the level of consciousness to two binary variables (RCONSC1 $=$ drowsy/alert and RCONSC2 $=$ unconscious/ alert) as in [23]. We follow the same steps as the steps in the simulation data application, optimizing the parameters of the pre-transformations and then training the VAE. Table 2 shows the quantitative comparisons. For the real data, we see that the QP-VAE is performing the best followed by our proposed approach and the Norta-j, which outperforms our method in $\bar{\psi }$ and it performs worse in the $\psi _{ratio}$. In general, as the data structure becomes more complex, the ability of CART to accurately distinguish between synthetic and original data can fluctuate more, leading to greater variability in $\psi$. This increased variability reflects the challenges of capturing complicated patterns in the data and is further amplified when using the $\psi _{ratio}$ metric. Therefore, it is expected that moving from a simulation data to a real example data, we have higher variability in $\psi _{ratio}$ using Norta-J, our approach, standard VAE, and QVAE. For GANs, we observe less variability, which, in combination with a higher mean, reflects a consistent level of poor performance in capturing the data complexities. This consistency suggests that GANs tend to generalize rather than specialize, leading to stable, though potentially less detailed synthetic data. The decision trees are fitted with a minimum leaf size of 20 and a maximum depth of 25.

In addition to quantitative comparisons, Fig. 3 shows the visual comparisons of the marginal density diagrams. Again, in this step, we used all the data points to generate the synthetic data. Here, we show three continuous variables, which exist in the dataset by baselines in comparison to the original data. As illustrated, only the VAEs with pre-transformation can generate the bimodality of the RDELAY variable in the data in contrast to the other variations of the VAE and the GAN, which generate an unimodal distribution. For the bimodality, the QP-VAE reconstructs the modes better than our proposed pre-transformation, but it still cannot generate a realistic range of data for the skewed distributions. For the real data, Norta-J cannot generate severe skewness as well as our approach, while it is successful in slight skewness. The other methods fail in the generation of skewed distributions. We use different sets of hyperparameters for the deep learning-based approaches and pick the most robust results.

**Fig. 4:**
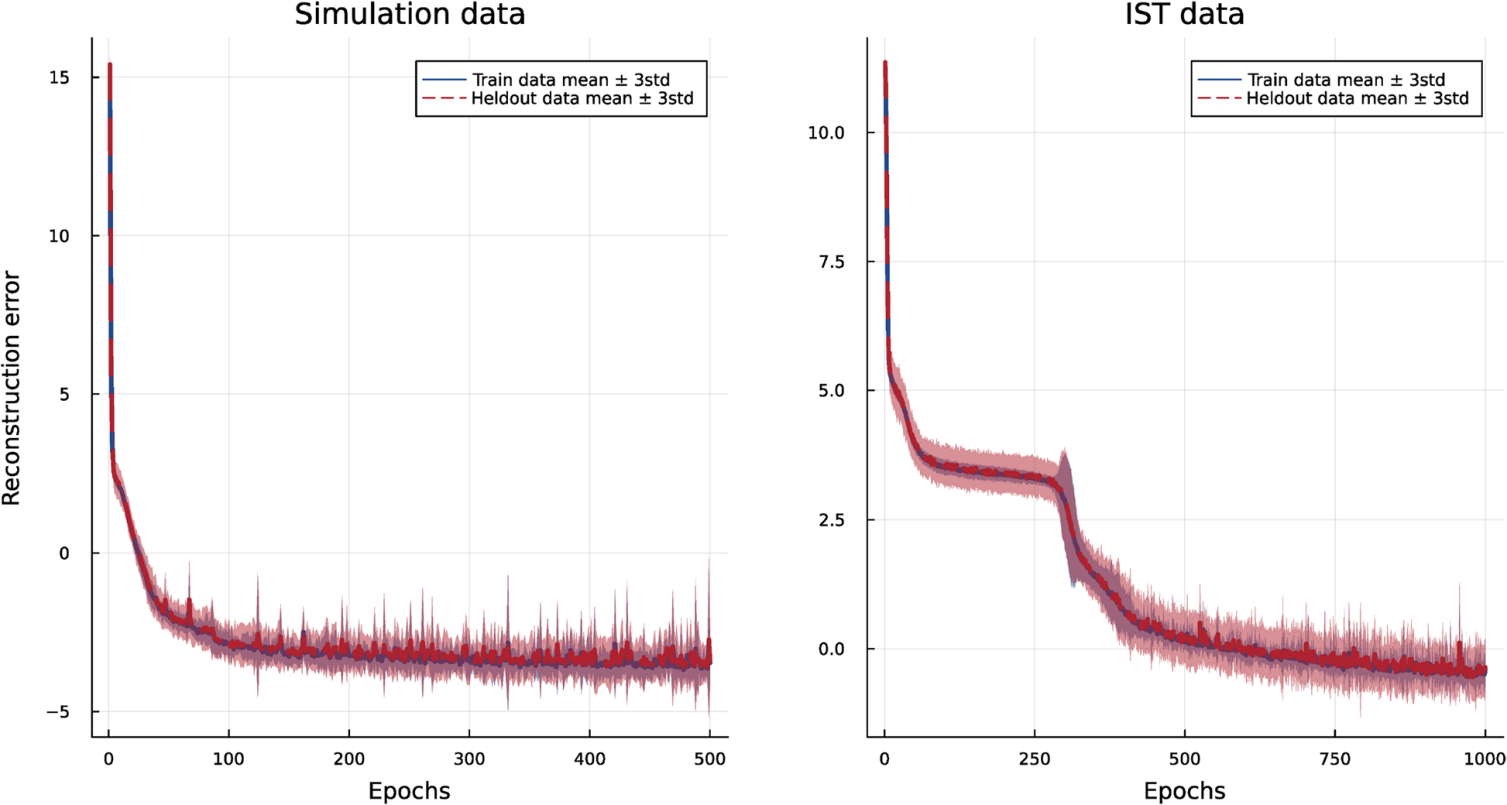
Mean and standard deviation of the reconstruction loss during the training of our VAE, plotted for both training and heldout data across 10 different folds. This illustrates the robustness of our approach by showing consistent performance across training data and heldout data

#### Robustness

As discussed in “Simulation data” section, we applied the 10-fold cross-validation method to both datasets and reported the uncertainty metrics for all the evaluated methods. To demonstrate the robustness of our approach, we have plotted the mean and standard deviation of the reconstruction loss during the VAE training process for both the training data and the heldout data across ten different folds, as shown in Fig. 4. In both data scenarios, the behavior of the model for unseen data and training data is very consistent. In some parts of the curve for the IST data, we see a smaller standard deviation (more stability) for the training data than the heldout data, which is a natural behavior.

## Evaluation of the method for known sub-group structures

### Simulation data

**Fig. 5:**
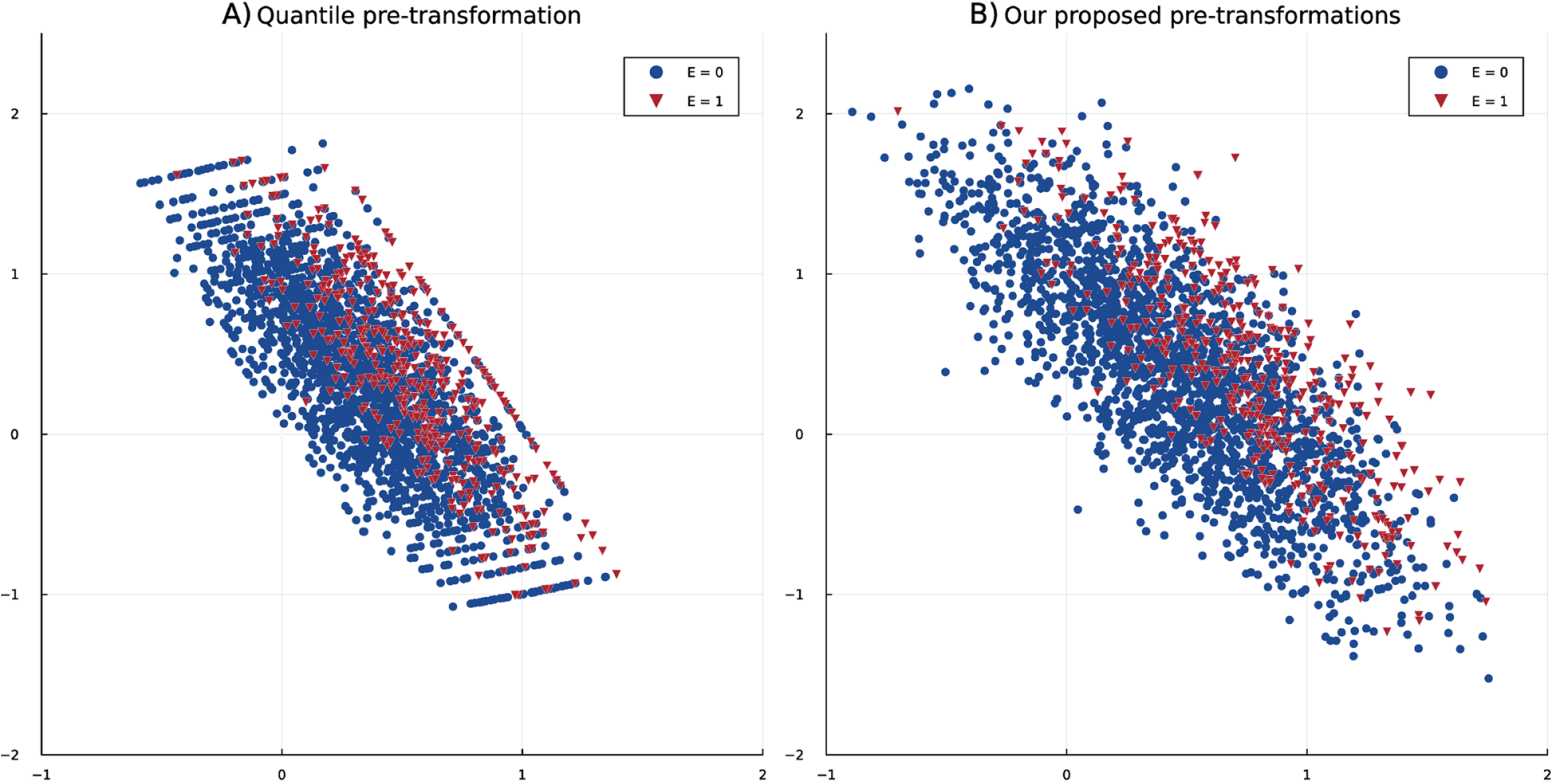
The latent representation of simulation design removing the confounding variable. **A** shows the latent structure learned by VAE when applying the quantile transformation, and **B** is the visualization of latent space when using our proposed pre-transformations. In both plots, the red triangles denote the exposed individuals, and the blue circles symbolize the non-exposed individuals

We start with the simulation design to explore the possibility of integrating propensity scores with the latent representation of VAE. In this step we investigate the pre-transformation VAEs for their capabilities of building a meaningful latent structure. Figure 5 shows the two-dimensional latent structure produced by the VAEs following the quantile transformation (A) and our proposed pre-transformations (B). We see that the quantile structure is reflected in the latent representation learned by VAE, i.e., the latent structure is not as smooth as our approach, especially at the edges. These discontinuities can make extracting meaningful features or patterns from the latent representations harder. This, in addition to the privacy issue and compromised fairness in the data, makes the QP-VAE less effective for generating synthetic data in presence of sub-groups. To investigate the integration of propensity scores with the latent representation of VAE, we can use a validity check based on our previous study [15]. Since variable selection is one of the challenging steps of propensity score model building, in the previous study, we investigated the simulation design to see whether the variables should be selected in relation to exposure alone, outcome alone, exposure and outcome, or both exposure and outcome. In this use case, we concluded that selecting variables directly related to exposure for this breast cancer-based simulation study gives more reliable results for estimating the propensity score. To inspect whether the latent representation of the VAE also captures these patterns, we overlay a heat map based on the propensity score grid with a scatterplot of the latent representation color-coded by two cohorts (exposed and non-exposed), and the value of the outcome variable is differentiated by shape. If the color patterns from the propensity scores, calculated with variables related to exposure, align better with the color of data points in the latent space, in comparison to the variable selection considering both exposure and outcome, it confirms our previous conclusion. This would suggest that our methodology is indeed promising for guided prior sampling. For training the VAE, we excluded the exposure and outcome variables. Moreover, we excluded the variable $x_6$, corresponding to the progesterone receptor status, because the data is simulated such that $x_6$ is related to both exposure and outcome and can be approximated by other variables. So, this way, we can have a scenario that has an unmeasured confounder. Then, we can investigate whether the propensity score-based values match the latent structure and check if the latent structure corresponds to the mentioned results in [15].

**Fig. 6:**
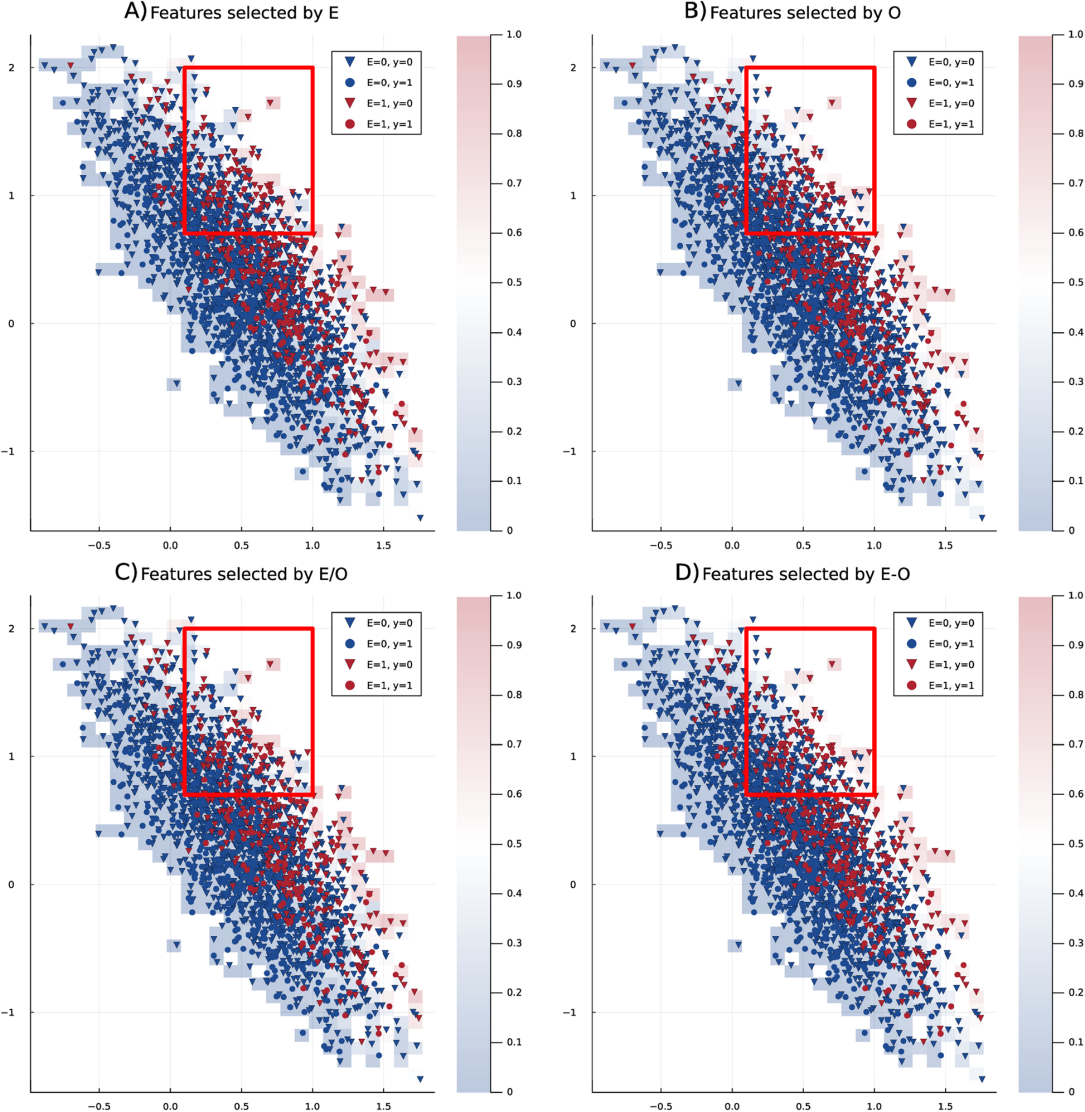
Latent representation of the simulation design extracted by a Variational Autoencoder (VAE), with the confounding variable *x*6 removed. Blue points represent the non-exposed cohort, while red points indicate the exposed cohort. Circles denote individuals who experienced the outcome, and triangles represent those without the outcome. **A** shows the heat map color-coded based on the propensity score, which is calculated by a selection of variables related to exposure, **B** the heat map color-coded based on the propensity score, which is calculated by a selection of variables related to outcome, **C** the heat map color-coded based on the propensity score, which is calculated by a selection of variables related to both exposure or outcome and **D** the heat map color-coded based on the propensity score, which is calculated by a selection of variables related to exposure and outcome. The area outlined by the red square shows the most important differences between the four variable selection methods

Then, we use logistic regression on the original variables to predict the exposure and selected variables related to exposure if their *p*-value was smaller than 0.05. Then, we selected variables related to the outcome by applying the logistic regression on the original data for predicting the outcome and including variables with a *p*-value smaller than 0.05. Then, we fitted four models. In Fig. 6, we see that regardless of the variable selection method, the general structure of latent space matches the propensity score-based values reflected in the colored grid behind the latent representation. Moreover, the area outlined by the red square shows that as this area has more blue data points, i.e., representing the non-exposed individuals, the propensity score model, which generates more blue grid cells would be the better approach. In Fig. 6, we see that the model with variables related to exposure and the E/O model, i.e., the selected variables are the union of variables related to outcome and variables related to exposure, are very similar and show a better match. Since the first model has fewer parameters, the exposure-only approach is preferred. Therefore, the results align with our previous study, which found that the model with variable selection directly related to exposure is the better variable selection method for this dataset. With this, we can conclude that combining propensity score regression with VAEs can be a promising sampling guide for VAEs.

### Real data

**Fig. 7:**
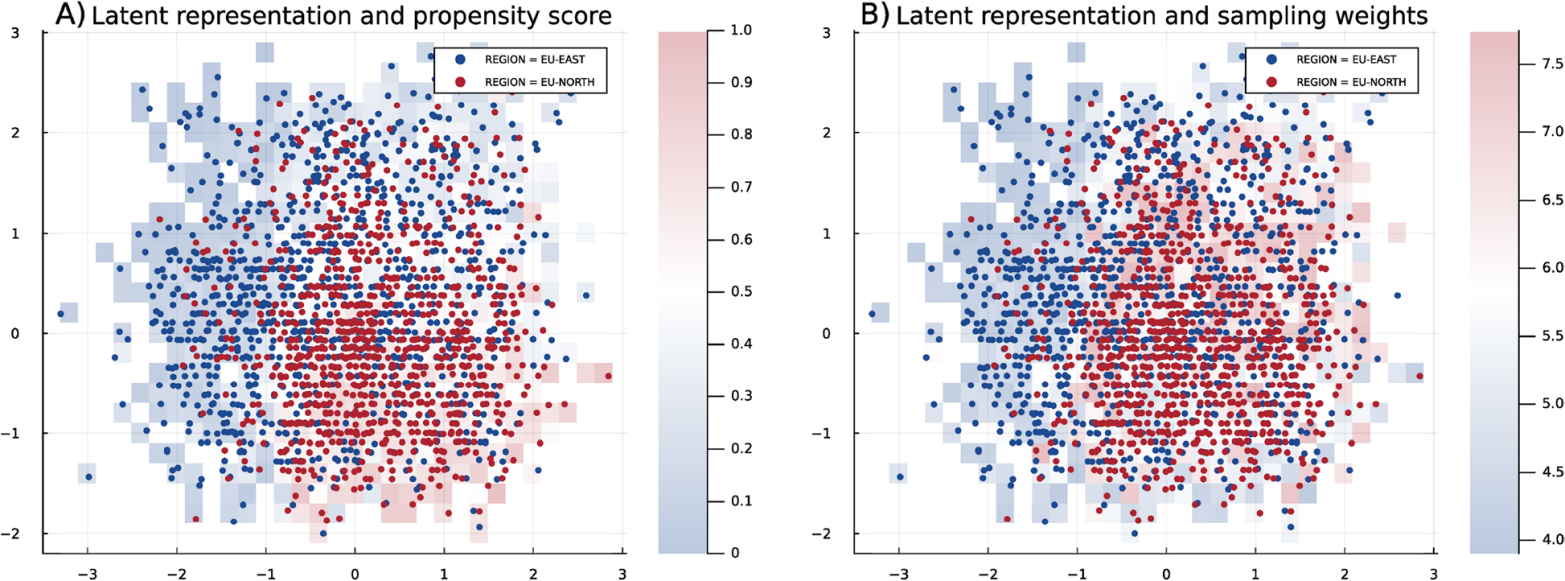
The latent representation of IST data, extracted by VAE. In both **A** and **B**, the blue dots represent the observations that belong to the region EU-EAST, and the red dots represent the observations that belong to EU-NORTH. In A, the heat map is color-coded based on the averaged propensity score, which is calculated by variable selection related to the region. In B, the heat map is color-coded based on the calculated weights for prior sampling when the target scenario is to remove the systematic differences

In the real data example, the sub-groups are related to the moderating variable of region membership, since it effects in different ways, e.g., variables related to the healthcare system or population-specific characteristics. Therefore, we use the REGION variable for the propensity score, fitting the logistic regression on original values predicting the REGION (EU-NORTH $= 1$ and EU-EAST $= 0$), and we select variables according to *p*-value with the cutoff $\alpha$ set to 0.05. Then, using the weighting approach for generating individuals common for both sub-groups from Eq. (14), we calculate the weights for the weighted sampling from the prior explained in “Propensity score-based sampling method” section. Getting the latent representation from the trained VAE, explained in “Evaluation of the method for unknown sub-group structures”, and overlaying the propensity score heat map and weight heat map, we obtain Fig. 7. The left plot in the figure confirms the feasibility of combining propensity score regression with the latent representation of VAE, as the areas with a majority of red dots correspond to the red grid cells. In the right plot, the red grid cells correspond to the areas with larger weights, i.e., with less systematic differences between the two sub-groups, and the blue grid cells correspond to the areas with sub-group-specific characteristics.

**Fig. 8:**
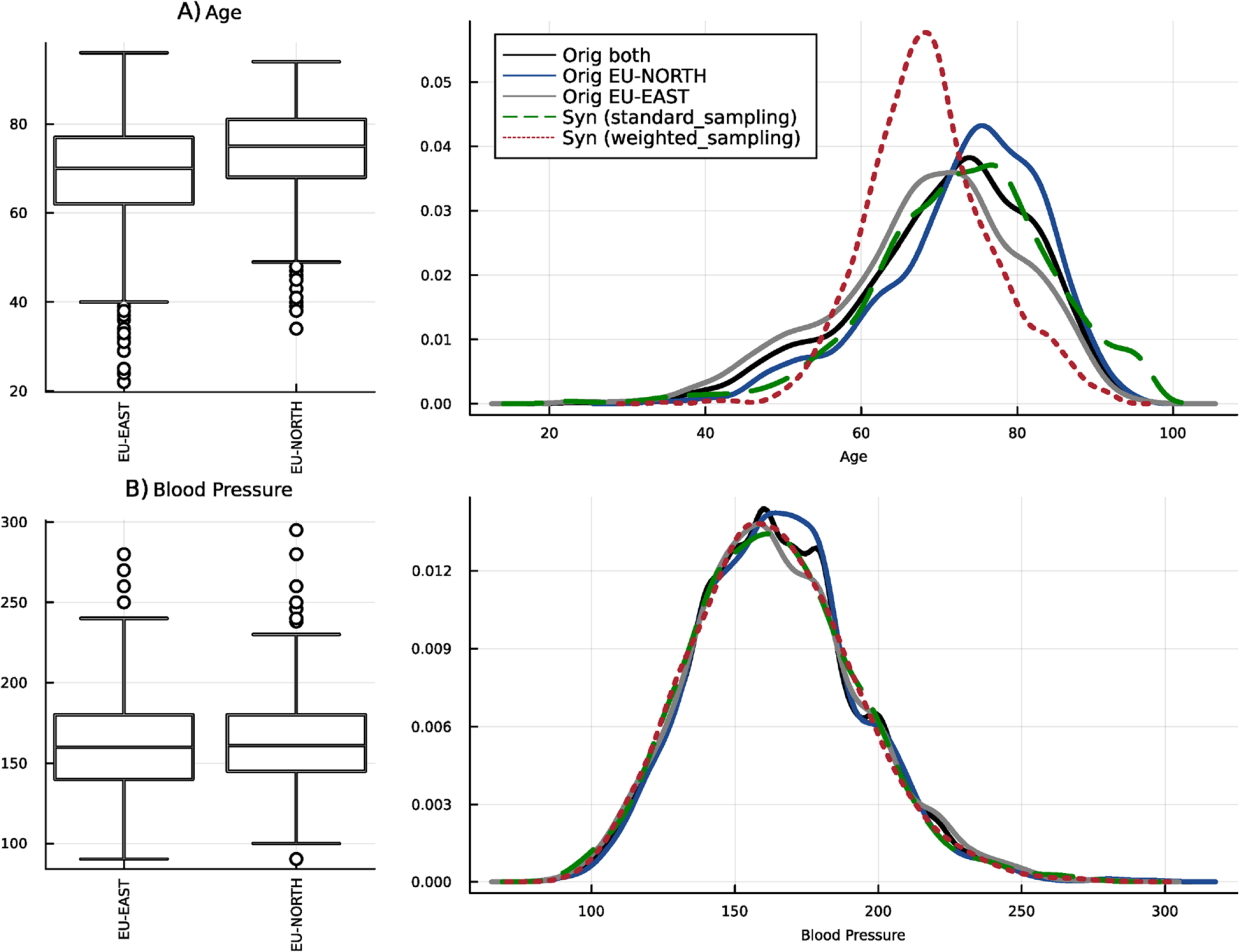
Visual comparisons of marginal distributions in synthetic dataset generated by different methods. This figure shows three continuous variables, including a bimodal distribution, i.e., shown in the first row. In the columns, different methods are illustrated

To investigate the impact of our approach, we compare marginal distributions from the two populations and generate data using standard and weighted sampling approaches. For this, we choose blood pressure, which has a similar distribution across the regions, and age, which is differently distributed, e.g., with a higher age of stroke in the EU-NORTH population. So, in this specific scenario, removing systematic differences means that in the synthetic data, we should not have a very high frequency of older individuals. The red dashed line in Fig. 8.B for the blood pressure variable shows that our approach recognizes no systematic differences for this variable. Therefore, the generated data has the same marginal distributions in both sub-groups. Still, when it comes to age, the marginal distribution is completely different (red dashed line in Fig. 8A, having a higher peak but almost similar mode to EU-EAST. The explanation for this is that because of differences in the population or in the healthcare system, EU-NORTH has a different underlying distribution. Getting back to the latent structure in Fig. 7B, the areas with blue grid cells, i.e., with smaller weights, have a higher concentration of EU-NORTH members. Therefore, with weighted sampling, we have fewer samples from those areas and can ensure that we do not have, e.g., many individuals with stroke age of 80 and generate a population that is on average younger than EU-NORTH.

For this result, we set the threshold for a zero weight $\delta$ from Eq. (14) to 0.1. Lower values, $\delta \approx 0$ are suitable for the scenarios where we are interested in preferentially sampling from the areas that have a rather similar group membership probability, while for higher values of $\delta$, we include samples which may be more common to one sub-group but still can be found in other sub-group as well. The heuristic approach of choosing the proper value is done using the visualization of the latent space structure. When $\delta$ is too large, we would have limited areas of interest, and if it is too small, most of the grid cells are included in the sampling. Overall, the results show that the weighted sampling approach is helpful when dealing with known sub-groups.

It is important to note the architectural choices of our model. For both of our datasets, we used a simple VAE. That is because when the model is more complex, e.g., having a higher-dimensional latent space or deeper architecture, it may overfit the training data. This overfitting can lead to the model memorizing specific details of the training data rather than learning a generalized representation. As a result, the synthetic data generated by the VAE might closely resemble the training data, leading to potential data disclosure issues. Additionally, as discussed in “VAE for combining continuous and binary variables” section, we tested both early and late fusion strategies as a hyperparameter. In our experiments, late fusion-using separate encoders for binary and continuous variables and averaging the latent space-yielded better latent structure and more realistic marginal distributions.

## Discussion

Variational autoencoders (VAEs) have shown promising results for generating image data, which is often evaluated based on the overall visual impression without analyzing individual pixel distributions. In contrast, synthetic clinical cohort data has different requirements, as heterogeneity is often a critical characteristic. Heterogeneity may be due to known sub-groups, e.g., reflecting different study sites, or may be unknown and just be reflected in marginal distributions. We investigated whether combining deep learning and classical statistical approaches — specifically pre-transformations for addressing heterogeneity reflected in bimodal or skewed distributions and propensity score regression for addressing known sub-groups — might be useful for synthetic data generation.

We used a realistic simulation based on a breast cancer study and a real international stroke dataset and showed that the proposed pre-transformation of the data can help reconstruct the complex marginal distributions, thus preserving the unknown sub-group structure. We compared our method with different baseline methods, among which QP-VAE (the non-parametric quantile transformation) showed strong performance in terms of quantitative metrics. Therefore, while QP-VAE has the important weakness of potential data disclosure risk, it can still be useful when the goal of synthesizing data is for data augmentation. In particular, it can be interesting for future work to improve this approach by first, increasing the fairness, i.e., adding the possibility of reproducing the outliers, and second, using the extensions that can handle out-of-distribution values. It is important to note that we need a higher number of quantiles (not appropriate for privacy-preserving scenarios) to have a smooth latent space using this pre-transformation. Despite these limitations, QP-VAE is a simple, non-parametric pre-transformation approach, which makes it a suitable option for data augmentation. For the known sub-groups, to see if propensity score estimation on the original data space can complement the VAE approach, we considered visualization in the latent VAE representation and found that propensity scores add complementary information. We illustrated the approach with a real dataset from an international stroke trial. The results show that our approach can reconstruct the more complicated marginal distributions, such as bimodal ones, even in the presence of different categorical/binary variables. We could obtain a latent representation that was useful for subsequent propensity score-guided sampling. Thus, extremes of sub-groups could be avoided in synthetic data.

Certainly, the proposed approach cannot address all potential types of heterogeneity, as we focused on bimodal and skewed marginal distributions, i.e., there might be other complex distributions that our approach cannot recover completely. Yet, these two are the most common marginal distributions in biomedical settings. Moreover, for the moment, we only focused on tabular data, but in clinical applications, such data may come in combination with other modalities like image data, and it needs specific considerations. Therefore, future work will need to investigate how to effectively integrate our approach with image data. Regarding the known sub-groups, we so far have not optimized the propensity score model, despite known challenges in model building [39]. Consequently, the proposed approach could probably be improved, e.g., by more closely investigating variable selection approaches for constructing the propensity score.

To summarize, the proposed approach illustrates that it can be useful to complement VAEs with more classical statistical modeling approaches for addressing heterogeneity when generating synthetic data. This can more generally pave the way for high-quality synthetic clinical cohort data in presence of sub-groups.

## Data Availability

Both datasets are publicly available. The original real data for the method comparison can be fetched from here, and the simulation design is available on Zenodo [37]. The pre-processed data can be found on the GitHub repository that also contains the complete reproduction script for the experiments.
